# Effectiveness of seasonal malaria chemoprevention administered in a mass campaign in the Kedougou region of Senegal in 2016: a case-control study

**DOI:** 10.12688/wellcomeopenres.18057.2

**Published:** 2022-09-30

**Authors:** Isaac Akhenaton Manga, Fassiatou Tairou, Amadou Seck, Ekoue Kouevidjin, Khadime Sylla, Doudou Sow, Alioune Babara Gueye, Mady Ba, Magatte Ndiaye, Roger Clément Kouly Tine, Omar Gaye, Babacar Faye, Jean Louis Abdourahim Ndiaye

**Affiliations:** 1 Department of Parasitology-Mycology/Faculty of medicine, pharmacy and odontology, University of Cheikh Anta Diop, Dakar, Senegal; 2 Ministry of Health and Social Action, National Malaria Control Program, Dakar, Senegal; 3 Service of Parasitology Mycology/Departement of medical biology, UFR Santé/University Iba Der Thiam, Thies, Senegal

**Keywords:** Seasonal malaria chemoprevention, Effectiveness, Case-control study, Senegal

## Abstract

**Background:** Seasonal malaria chemoprevention (SMC) with sulfadoxine-pyrimethamine plus amodiaquine (SPAQ) is a malaria prevention strategy recommended since 2012 by the World Health Organization (WHO) for children under 5 years. In Senegal, the scaling up of SMC started in 2013 in the south-eastern regions of the country with an extension of the target to 10 years old children. The scaling up of SMC requires regular evaluation of the strategy as recommended by the WHO. This study was conducted to evaluate the effectiveness of SMC.

**Methods:** A case-control study was conducted in some villages of the health districts of Saraya and Kedougou in the Kedougou region from July to December 2016. A case was a sick child, aged 3 months to 10 years, seen in consultation and with a positive malaria rapid diagnostic test (RDT). The control was a child of the same age group with a negative RDT and living in the same compound as the case or in a neighbouring compound. Each case was matched with two controls. Exposure to SMC was assessed by interviewing the mothers/caretakers and by checking the SMC administration card.

**Results:** Overall, 492 children, including 164 cases and 328 controls, were recruited in our study. Their mean ages were 5.32 (+/- 2.15) and 4.44 (+/-2.25) years for cases and controls, respectively. The number of boys was higher in both cases (55.49%; CI 95%=47.54-63.24%) and controls (51,22%; CI 95%=45.83-56.58%). Net ownership was 85.80% among cases and 90.85% among controls (p=0,053). The proportion of controls who received SMC was higher than that of cases (98.17% vs 85.98% and p=1.10 ^-7^). The protective effectiveness of SMC was 89% (OR= 0.12 (CI 95%=0.04-0.28)).

**Conclusions:** SMC is an effective strategy in the control of malaria in children. Case-control studies are a good approach for monitoring the efficacy of drugs administered during SMC.

## List of abbreviations

AQ             Amodiaquine

CI              Confidence interval

CHWs       Community health workers

D2             Day 2

D3             Day 3

HBCP        Home-based care provider

LLIN         Long-lasting impregnated mosquito net

OR            Odd Ratio

RDT          Rapid Diagnostic Test

SMC         Seasonal malaria chemoprevention

SP             Sulfadoxine-Pyrimethamine

SPAQ)      Sulfadoxine-Pyrimethamine plus Amodiaquine

TBS          Thin blood smear

TDS          Thick drop slide

WBC        White blood cells

WHO        World Health Organization

## Introduction

Seasonal malaria chemoprevention (SMC) is a strategy for malaria prevention in children under 5 years of age living in areas of moderate to high malaria transmission in sub‐Saharan Africa ^1^. It consists of intermittent full treatment with an antimalarial drug during the season of high malaria transmission to prevent the disease, with the objective of maintaining therapeutic levels of antimalarial drug in the blood during the period when the risk of contracting malaria is the highest ( WHO. Report of the Technical consultation on SMC, 2011, Implementation of Seasonal Malaria Chemoprevention: A report of two meetings). One single dose of Sulfadoxine-Pyrimethamine (SP) and 3 daily doses of amodiaquine (AQ) are administered monthly to obtain the preventive dose ^1– 3^. The drugs should be administered from the beginning of the transmission season, up to a maximum of four monthly cycles ^1^. The effectiveness of SMC has been demonstrated by numerous studies, which have also shown that it is well tolerated and inexpensive ^4– 7^ ( NMCP. Epidemiological report, 2019). These studies, conducted mostly between 2002 and 2011, showed that SMC would have prevented about 75% of all uncomplicated malaria attacks and also about 75% of severe malaria attacks. It would also have reduced child mortality by about 1 per 1000 and reduced the incidence of moderate anemia ^1, 4– 7^. Then SMC has been well received as a new tool offering a high degree of personal protection at a moderate cost ^4^ and was recommended since 2012 by the World Health Organization (WHO) as an additional prevention strategy for malaria control ^1^. Senegal, like many African countries in the south of the Sahara ^4^, has adopted and implemented SMC on a large scale since 2013 in the regions of the country, that are eligible according to WHO implementation criteria. SMC is administered through a door-to-door strategy based on the community system ^8– 10^. In Senegal, the SMC target was children aged 3 months to 10 years because numerous in-country and operational research studies on intermittent preventive treatment of malaria in children and SMC had shown that children 5–10 years of age were just as vulnerable as those under 5 ^5– 7^.

While recommending the implementation of SMC on a large scale, WHO also specifies the need to monitor several parameters such as pharmacovigilance, coverage rate, malaria morbidity and mortality, and the appearance of drug-resistant strains of parasites ^1, 4^. This study was therefore conducted to assess the effectiveness of SMC campaign in Senegal, using a case-control study.

## Methods

### Study site

**Figure 1.**
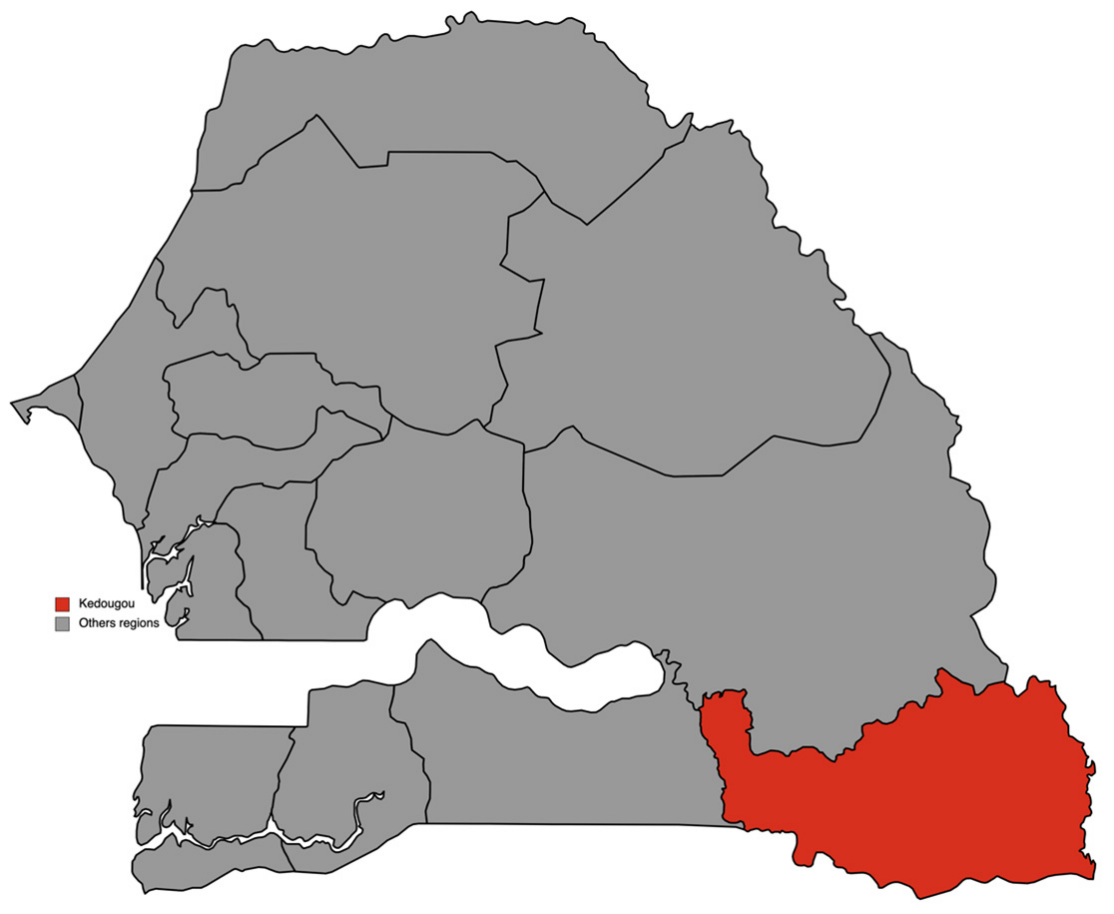
Map of region of Kédougou in Senegal. (This figure was made by the author for this paper using mapchart.net). This work is licensed under a Creative Commons Attribution-ShareAlike 4.0 International License.

This study took place in the region of Kédougou, at 700 km from Dakar, the country's capital. Located on the banks of the Gambia River, Kédougou is in the extreme southeast of Senegal and borders Mali and Guinea. This region is characterized by a sahelian climate with an average temperature of 29.3°C and an average rainfall of 926.2 mm. It includes three departments (Kédougou, Salémata and Saraya) corresponding to the three health districts of the region ( Figure 1). Malaria in Kédougou is a real public health problem because, in 2019 for example, the proportional malaria morbidity was 27%, the rate of test positivity (RDT and blood smear) in the general population was 51%, in children under 5 years of age this rate was 26%. The proportional malaria mortality in this region was 27% and 50% in children under 5 years of age ( NMCP. Epidemiological report, 2019, ^6^). These different conditions made this region eligible for SMC, which has been implemented there since 2013. This study was conducted in villages with either a head nurse, or with a community health worker (CHW), or also a DSDOM (Home health care provider) in the health districts of Kédougou and Saraya ( Figure 1) ^11^.

### Study type, time period, and population

A case-control study was conducted from July to December 2016. Assuming a two-sided confidence level of 95% with a power of 80% and a match of one case to two controls, and a percentage of exposed cases of approximately 50%, the Epi info 7.1.3.3 software (RRID:SCR_021682) estimated our study population at 152 cases and 304 controls. Being between 0 to 10 years of age, residing in our study sites and for whom the parents had given free and informed written consent, were the main inclusion criteria for this study. Any child who met the inclusion criteria, self-referred to a health facility in the study site and had a positive rapid diagnostic test (RDT) for malaria was considered as a “case”. The "control" was a child of the same age group, living in the same compound or in a neighboring compound within 10 meters. Controls were recruited at concession level based on an apparent good health (without any clinical symptom) and a negative RDT. Each case was matched with two controls.

### Conduct of the study

The purpose and objectives of the study were first shared with the health authorities in the region, prior to the training of the field staff including community health workers (CHWs) and the head nurses for data collection. Each case and control will therefore be visited at home to record the current level of mosquito bed net use (based on inspection of where the child sleeps, the type and condition of the net at the time of case detection); other interventions like the rate of SMC dosing; and the coverage of mosquito bed net use and other protective measures at concessions in the vicinity of the person's home.

A capillary blood sample was also taken from the pulp of the finger from each subject included in the study, for a rapid diagnostic test (RDT) and the preparation of a thick and a thin blood smear. The slides were stained for 15 minutes with a 10% Giemsa R solution (RAL, REF: 320310-2500; LOT: 037834) and then read by technicians from two different facilities. The slides were read at objective 100 with immersion oil on LEICA DM500 microscopes. Parasite density was assessed by counting the number of asexual parasites per 200 white blood cells (WBC) and estimated by the number of parasites per µl using the following formula: number of parasites × 8,000/200 assuming a WBC count of 8,000 cells/µl. Thick and thin blood smears were considered negative after microscopic reading of 100 fields with no parasites detected. Their reading was done according to the recommendations of the national guidelines for biological diagnosis of malaria in the laboratory ( NMCP. National diagnostic guidelines for malaria, 2018.).

### Data management and analysis

The different questionnaires and biological results were entered on a data entry mask developed with Microsoft Excel 2019, 16.60 (22041000) (RRID:SCR_016137) software. Data was analyzed with Epi Info 7.1.3.3 software (RRID:SCR_021682). Quantitative variables were described in means and standard deviation. Inter-group comparisons were made using the ANOVA test or Student's t-test according to the conditions of application of these tests. When these tests were not applicable, nonparametric tests (Mann Witney, Kruskall Wallis) were used. Categorical variables were presented in percentage with confidence interval (CI). Proportions were compared using Chi-square test or Fisher exact test (univariate analysis). Risk factors were assessed by multivariate survey logistic regression models. The significance level of the different tests was 0.05 two-tailed. The effectiveness of the different malaria prevention methods investigated in this study was calculated using the following formula: Efficiency =1-OR ( Médecin sans frontière. Efficacité vaccinale | Guides médicaux). Only patients included in the study were taken into account in the data analysis.

### Ethics considerations

This study received in October 2013 approval from the National Health Research Ethics Committee of Senegal under the number CNERS SEN13/57. Informed written consent from the parents or legal representative was a prerequisite for inclusion in the study. In order to respect confidentiality, an identification code was given to each participant.

## Results

### Socio-demographic characteristics

A total of 492 children aged 4 months to 10 years with a mean age of 4.73 (+/- 2.25) years were recruited in this study ^11^. They consisted of 164 cases and 328 controls. The number of boys was higher in both cases (55.49%; CI 95%=47.54-63.24%) and controls (51,22%; CI 95%=45.83-56.58%) and there was no statistically significant difference compared to those of the opposite sex (p=0.1868). Recruitment was as follows for cases: 1.82% (CI 95%=0.38-5.25%) in July; 29.87% (CI 95%=22.99-37.51%) in August; 26.22% (CI 95%=19.67-33.65%) in September; 22.56% (CI 95%=16.41-29.73%) in October; 15.85% (CI 95%=10.63-22.36%) in November and 3.66% (CI 95%=1.35-7.79%) in December. For controls, there was 0.6% (CI 95%=0.17-2.20%) in July; 28.96% (CI 95%=24.32-34.09%) in August; 22.86% (CI 95%=18.65-27.71%) in September; 21.65% (CI 95%=17.53-26.42%) in October; 19.82% (CI 95%=15.86-24.47%) in November and 6.10% (CI 95%=3.98-9.23%) in December. However, there was no relationship between the period of recruitment of cases and controls (p=0.5009) ( Table 1) ^11^.

**Table 1.** Distribution of the study population according to sociodemographic characteristics.

	Case (N=164)	Control (N=328)
**Sex**		
• **Boys**	91 (55.49% ; CI 95%=47.54-63.24%)	168 (51,22% ; CI 95%=45.83-56.58%)
• **Girls**	73 (44.51% ; CI 95%=36.76-52.46%)	160 (48.78% ; CI 95%=43.42-54.17%)
**Recruitment period**		
• **July**	3 (1.82% ; CI 95%=0.38-5.25%)	2 (0.6% ; CI 95%=0.17-2.20%)
• **August**	49 (29.87% ; CI 95%=22.99-37.51%)	95 (28.96% ; CI 95%=24.32-34.09%)
• **September**	43 (26.22% ; CI 95%=19.67-33.65%)	75 (22.86% ; CI 95%=18.65-27.71%)
• **October**	37 (22.56% ; CI 95%=16.41-29.73%)	71 (21.65% ; CI 95%=17.53-26.42%)
• **November**	26 (15.85% ; CI 95%=10.63-22.36%)	65 (19.82% ; CI 95%=15.86-24.47%)
• **December**	6 (3.66% ; CI 95%=1.35-7.79%)	20 (6.10% ; CI 95%=3.98-9.23%)

Thick blood count was positive in 87.80% (CI 95%=81.8-92.39%) of cases and 2.74% (CI 95%=1.45-5.135%) of controls. There was an association between the result of the thick blood test and whether the child was a case or a control (chi-square-corrected (Yates)=365.23 and p=1.10 ^-9^). Plasmodium falciparum was the only species found on positive slides for both cases and controls. The mean parasite density was 13820.18 (+/-19393.43) in cases versus 4119.66 (+/- 3827.74) in controls and this difference was statistically significant (T-test=4,71 and p=1.10 ^-4^).

### Malaria prevention

***Mosquito net.*** Net ownership was much higher among controls (90,85%; CI 95%=87.24-93.52%) than among cases (85,80%; CI 95%=79.7-90.9%) and this difference was not statistically significant (chi-square-corrected (Yates)=2.22 and p=0.13). The rate of net use was higher among controls who had slept under a net the day before the survey (99.65%; CI 95%=98.06-99.99%) compared to cases (96.24%; CI 95%=91.44-98.77%). This difference was statistically significant (chi-square-corrected (Yates)=5.23 and p=0.007). Comparing the possession or not of a long-lasting impregnated mosquito net (LLIN) in cases and controls, an odds ratio of 0.61 (CI 95%=0.34-1.10) was found. This gives an effectiveness of the LLIN of 39% in this study.

***Exposure to SMC.*** This study has also assessed the use of SMC among case and controls. It was reported that the controls (98.17%; CI 95%=96.07-99.16%) had taken more SMC than the cases (85.98%; CI 95%=79.7-90.9%) and this difference of proportion was statistically significant (chi-square-corrected (Yates)=27.15 and p=1.10 ^-7^). Comparing the use or not of SMC between cases and controls, an odds ratio of 0.12 (CI 95%=0.04-0.28) was found. This gives an effectiveness of 88% to this strategy. Of the cases who received SMC, 68.38% (CI 95%=59.86-76.08%) were recruited after less than 28 days from the last time they took SMC, 27.94% (CI 95%=20.59-36.28%) between 29 and 42 days, and 3.68% (CI 95%=1.20-8.37%) more than 43 days. For controls who received the drug, 79.10% (CI 95%=74.24-83.25%) were recruited before 28 days, 18.65% (CI 95%=14.71-23.35%) between 29 and 42 days, and 2.25% (CI 95%=1.09-4.57%) after more than 43 days.

There was no statistically significant difference between cases and controls, regardless of the time period between the date of the last administration of SMC and the date of recruitment (with the Fisher’s exact, p=0.05). Among the cases, 26.99% (CI 95%=20.35-34.5%) had not received any SMC cycle, 23.93% (CI 95%=17.60-31.22%) received one cycle; 21.47% (CI 95%=15.44-28.58%) two cycles; 16.56% (CI 95%=11.21-23.18%) three and 11.04% (CI 95%=6.68-16.89%) four. For controls, the proportions of children also varied according to the number of cycles received. Indeed, 4.27% (CI 95%=2.56-7.04%) had not received any; 22.56% (CI 95%=16.41-29.73%) one; 25.30% (CI 95%=20.9-30.28%) two; 24.09% (CI 95%=19.77-29%) three and 17.07% (CI 95%=13.39-21.52%) four cycles. These differences in proportions between these two groups of children according to the number of cycles received, were statistically significant (Chi-square=54.88 and p=1.10 ^-4^). In the case group, 82.55% (CI 95%=75.49-88.27%) of the children reported that the community health worker had left the doses of day 2 (D2) and day 3 (D3), compared to 98.45% (CI 95%=96.42-99.33%) of the controls. This difference was statistically significant (p=0.0000004). Compliance with these doses was more observed in the controls with 98.43% (CI 95%=96.38-99.33%) having taken the dose on D2 and 96.24% (CI 95%=93.54-97.84%) on D3. In the cases, the compliance was 86.51% (CI 95%=79.28-91.94%) for D2 and 73.02% (CI 95%=64.38-80.53%) for D3. There was a statistically significant difference between cases and controls for both D2 (Chi-square=27.89 and p=1.10 ^-4^) and D3 (Chi-square=54.43 and p=1.10 ^-4^). The number of controls who used both net and SMC (84.45%) was higher than that of cases (60.97%). This difference in proportion was statistically significant (p=0.032) ( Table 2) ^11^.

**Table 2.** Distribution of cases and controls according to the means of prevention (mosquito net and SMC) used. SMC = Seasonal malaria chemoprevention, LLIN= Long-lasting impregnated mosquito net, CHW= Community health worker.

	Cases (N=164)	Controls (N= 328)	P (p-value)
LLIN			
• Possession of a mosquito net	144 (85.80% ; CI 95%=79.7-90.9%)	298 (90.85% ; CI 95%=87.24-93.52%)	0.053
• Net use the day before the survey	128 (96.24% ; CI 95%=91.44-98.77%)	284 (99.65% ; CI 95%=98.06-99.99%)	0.007
SMC			
Taking of SMC	141 (85.98% ; CI 95%=79.7-90.9%)	322 (98.17% ; CI 95%=96.07-99.16%)	0,0000001
Last SMC			
• < 28 days	93 (68.38% ; CI 95%=59.86-76.08%)	246 (79.10% ; CI 95%=74.24-83.25%)	0.05
• 29–42 days	38 (27.94% ; CI 95%=20.59-36.28%)	58 (18.65% ; CI 95%=14.71-23.35%)	
• > 42 days	5 (3.68% ; CI 95%=1.20-8.37%)	7 (2.25% ; CI 95%=1.09-4.57%)	
Number of monthly treatments received			
▪ 0	44 (26.99% ; CI 95%=20.35-34.5%)	14 (4.27% ; CI 95%=2.56-7.04%)	0,0001
▪ 1	39 (23.93% ; CI 95%=17.60-31.22%)	96 (22.56% ; CI 95%=16.41-29.73%)	
▪ 2	35 (21.47% ; CI 95%=15.44-28.58%)	83 (25.30% ; CI 95%=20.9-30.28%)	
▪ 3	27 (16.56% ; CI 95%=11.21-23.18%)	79 (24.09% ; CI 95%=19.77-29%)	
▪ 4	18 (11.04% ; CI 95%=6.68-16.89%)	56 (17.07% ; CI 95%=13.39-21.52%)	
Tablets delivered by CHW for D2 and D3	123 (82.55% ; CI 95%=75.49-88.27%)	317 (98.45% ; CI 95%=96.42-99.33%)	4.10 ^-9^
Taking the tablet at D2	109 (86.51% ; CI 95%=79.28-91.94%)	314 (98.43% ; CI 95%=96.38-99.33%)	0.0001
Taking the tablet at D3	92 (73.02% ; CI 95%=64.38-80.53%)	307 (96.24% ; CI 95%=93.54-97.84%)	0.0001
LLIN and SMC	60.97% (100)	84.45% (277)	0,032

## Discussion

In this case-control study chosen to evaluate the effectiveness of SMC administered in a mass campaign in Senegal, the same difficulties as those described by Cairns *et al.*, and related to the rigor required for this type of study, were encountered ^12^. The usefulness of case-control studies for determining the efficacy of SMC as well as that of a vaccine has been reported. Indeed, this type of study would allow a better understanding of many parameters that could have an impact on it ^4^. Home visits to collect information were not facilitated by the rainy season, which sometimes made access to the villages difficult, but which was also linked to farming activities. This case-control study has resulted in a protective efficacy of 89% of the SMC not exceeding 28 days. Similar results, with an efficacy of 88% in the first 28 days. The same effectiveness were also almost obtained in a study that evaluated SMC in 5 West African countries where SMC was also implemented ^12^. The Access SMC consortium, which supervised the scaling up of SMC in West and Central Africa, also found during its evaluation that this strategy, similar to ours, was protective ^4, 13^. This very good efficiency of the SMC around 90%, had already been demonstrated in many studies conducted in the research context ^8^. This observation shows that the transition from research to scale-up of this strategy does not affect its effectiveness ^2, 12, 13^. However, it is strongly related to the complete treatment as demonstrated by this and several other studies ^8^.

In this study, the evaluation of the efficacy of the net was also conducted at the same time as the SMC. It was found that SMC was more effective than the net (89% vs 45%). The same observation was also made by Cairns *et al.,* in 2015 in Gambia (85% vs. 49.9% in 2015) ^12^. The efficacy of the net around 50% found in this study had also been showed in other studies that sought to evaluate. On the other hand, efficiencies higher than ours can also be noted ^9, 10, 12^.

In this study, controls had higher use of both SMC and nets. This indicates the need to strengthen advocacy for the integrated use of all malaria prevention strategies to have a greater impact on malaria indices ( NMCP. Epidemiological report, 2019.), ( NMCP. National strategic plan for malaria control in Senegal 2016-2020.) ^10^.

## Conclusion

This study showed that this strategy was very effective in preventing malaria in children. However, the sustainability of SMC should also include an evaluation of its efficacy *in vitro* and at the molecular level.

## Data Availability

Dryad: Effectiveness of seasonal malaria chemoprevention administered in a mass campaign in the Kedougou region of Senegal in 2016: a Case-control study. https://doi.org/10.5061/dryad.j9kd51cg6 ^11^. This project contains the following underlying data: Data file: Case-control 2016.xlsx This project contains the following extended data: CRF case-control.pdf map_of_Kedougou_region.pdf Negative_slide_of_thin.jpeg Positive_slide_with_P._falciparum_in_the_middle.png Data are available under the terms of the Creative Commons Zero “No rights reserved” data waiver (CC0 1.0 Public domain dedication).
