# Supplementary material for: Effectiveness of seasonal malaria chemoprevention administered in a mass campaign in the Kedougou region of Senegal in 2016: a case-control study
Source: Wellcome Open Res. 2023 Apr 12;7:216. Originally published 2022 Aug 19. [Version 3] doi: 10.12688/wellcomeopenres.18057.3 (PMC10154913; doi:10.12688/wellcomeopenres.18057.3)
Supplement: Peer review history

# Peer review: Reviewer response for version 3

**DOI:** 10.21956/wellcomeopenres.21227.r56180

**Published:** 2023-05-02

**Authors:** Prudence Hamade

**Affiliations:** 1 Malaria Consortium, London, UK

The authors have improved the article very well. It explains more clearly the results.

 The results are very interesting and I think the discussion could go into them a little more. For example, it would be interesting to know if there were any other confounding factors in the control group. The controls it seems both took the medication better and slept under nets more regularly. Was this because they were younger (slightly on average) or came from smaller families, had more conscientious parents, presence of a grandmother in the house older mothers vs. younger mothers better housing etc?

 It was also interesting that 68.38 % of cases had received some SMC and got malaria within 28 days. What were the associated factors for this? Even more interesting was that 11.04 % of cases had received 4 doses of SMC and 4.27% of controls had not received any SMC, were they older children who might have some immunity already? These are all future research questions perhaps but could be described as being limitations of this study. The conclusion might therefore add some opinion on the unknown. We have one of the know facts seen in this paper is that malaria prevalence is still high in the general population even if slightly lower in children under five years and that this will continue indefinitely unless resources are directed to driving down transmission as well as preventing infection for a short period every year in a defined age group.

Is the work clearly and accurately presented and does it cite the current literature?

Partly

If applicable, is the statistical analysis and its interpretation appropriate?

I cannot comment. A qualified statistician is required.

Are all the source data underlying the results available to ensure full reproducibility?

Yes

Is the study design appropriate and is the work technically sound?

Yes

Are the conclusions drawn adequately supported by the results?

Yes

Are sufficient details of methods and analysis provided to allow replication by others?

Yes

Reviewer Expertise:

Malaria and childhood disease

I confirm that I have read this submission and believe that I have an appropriate level of expertise to confirm that it is of an acceptable scientific standard.

# Peer review: Reviewer response for version 3

**DOI:** 10.21956/wellcomeopenres.21227.r56179

**Published:** 2023-04-13

**Authors:** Drissa Konaté

**Affiliations:** 1 Malaria Research and Training Center, Bamako, Mali

I am ok.

Is the work clearly and accurately presented and does it cite the current literature?

Yes

If applicable, is the statistical analysis and its interpretation appropriate?

Partly

Are all the source data underlying the results available to ensure full reproducibility?

Yes

Is the study design appropriate and is the work technically sound?

Yes

Are the conclusions drawn adequately supported by the results?

Yes

Are sufficient details of methods and analysis provided to allow replication by others?

Partly

Reviewer Expertise:

Epidemiology

I confirm that I have read this submission and believe that I have an appropriate level of expertise to confirm that it is of an acceptable scientific standard.

# Peer review: Reviewer response for version 2

**DOI:** 10.21956/wellcomeopenres.20402.r54533

**Published:** 2023-02-15

**Authors:** Prudence Hamade

**Affiliations:** 1 Malaria Consortium, London, UK

This is an interesting study with interesting results although the results only confirm numerous other cases control studies that have been done across the Sahel

1. It should be made clear when the scaling up to aged 10 years was done.
2. This study was done in 2016 why is it only being published now?
3. The odds ratios presented for both SMC and bed net usage efficacy are crude estimates in that they are not adjusted for confounding by each other. Adjusted (for confounding) odds ratios can be obtained by fitting a multivariable logistic regression model with both explanatory variables. Alternatively adjusted odds ratios could be estimated using stratification.
4. Be consistent with presentation of P values - give them to 4 decimal points only. If smaller than that then present as e.g. P < 0.0001.
5. *"Malaria in Kédougou is a real public health problem because, in 2019 for example, the proportional malaria morbidity was 27%, the rate of test positivity (RDT and blood smear) in the general population was 51%, in children under 5 years of age this rate was 26%. The proportional malaria mortality in this region was 27% and 50% in children under 5 years of age ( NMCP. Epidemiological report, 2019, ^6^)"*.
  These figures in themselves need a bit of explanation. Proportional malaria morbidity needs to be seen as a proportion of what Total morbidity. Total of suspected malaria cases or cases of fever etc. If malaria mortality in children under five is 50%. 50% of what? 50% of total under five mortality is still shocking in a country which has been implementing SMC for 6 years (2019) However has child mortality reduced significantly so the numbers of children dying from malaria may have been reduced even if proportionate mortality is still 50%

I am not sure what this study has to add to the evidence provided by several other case control studies that have been done to show the effectiveness / efficacy of SMC in reducing malaria cases in the Sahel. Its main difference is that it is conducted in children 3 months to 10 years (number of months) and that the average age of cases in this study was in slightly older children than would be expected if looking at traditional SMC in children from 3 - 59 months. New information of value in this study could have been if SMC was as effective in older children (those over five years) as in the younger age group which might have led to more programmes adopting the strategy of including older children which might in turn have a greater effect in the long run of reducing the malaria burden and leading down the road to actually eliminating malaria which from their data on malaria morbidity and mortality in 2019 was still very high in both the general population and in children under five. SMC it seems reduces morbidity in children especially if all four cycles are completed with 3 days of medication and the protective effect is best in the 28 days following the dose. If in spite of many years of implementation malaria transmission remains high, as it seems to here, we need to develop other strategies if we really want to have a serious effect on morbidity and morality over the long term as nets and SMC have not led to a reduction in transmission - perhaps this could be mentioned in the discussion even when extended to older children?

Is the work clearly and accurately presented and does it cite the current literature?

Partly

If applicable, is the statistical analysis and its interpretation appropriate?

I cannot comment. A qualified statistician is required.

Are all the source data underlying the results available to ensure full reproducibility?

Yes

Is the study design appropriate and is the work technically sound?

Yes

Are the conclusions drawn adequately supported by the results?

Yes

Are sufficient details of methods and analysis provided to allow replication by others?

Yes

Reviewer Expertise:

Malaria and childhood disease

I confirm that I have read this submission and believe that I have an appropriate level of expertise to confirm that it is of an acceptable scientific standard, however I have significant reservations, as outlined above.

# Response

**Published:** 2023-02-23

**Authors:** Isaac Akhenaton MANGA

**Affiliations:** Faculté de Médecine FMPO/UCAD, Senegal

1. In Senegal the target of SMC, since the beginning of its implementation and scaling up in 2013 the southern regions of the country, has been children aged 3 to 120 months. The expansion of the SMC target in Senegal was decided at the time the strategy was adopted by the country's health authorities. This choice was reinforced by the numerous studies on intermittent preventive treatment (IPT) of malaria in children, which had shown that children aged 5 to 10 years were just as vulnerable as those under 5 years. This idea was submitted to WHO, which endorsed it, thus enabling the country, with the support of its technical and financial partners, to implement SMC for this group age

 2. This study is part of a project to evaluate the impact of scaling up SMC in Senegal. This evaluation was requested from our organisation by the NMCP and was to be an ongoing process. However, it has not been carried out since 2016, although we had hoped to continue it in order to try to determine the dynamics of the effectiveness of the scaled-up SMC over time. This situation explains why it's took time before to write this article.

 3. We accept your recommendation to adjust the odd ratio before the efficiency calculation. We will do this and include it in the new version we submit to the journal.

 4.We have standardized the presentation of the p-value as you suggested. The changes have been made in the new version submitted to the journal.

 5. We have reworded the sentence because, as noted, it is confusing. We also think that the part about the burden of malaria in the proposed new version helps to present it better. Our study was conducted in children aged 3 to 120 years, unlike those conducted in the Sahel to determine the effectiveness of SMC, all of which, as you rightly pointed out, were targeted at children under the age of 5. We believe that even though we did not carry out an analysis to determine the effectiveness of this strategy in children under and over 5 years of age, this constitutes a strong point for our study. In addition, we believe that the other strength of this study is that the data collection (making mixed smear slides and recruiting both cases and controls, not to mention administering the questionnaires) was done by the trained community level and not by the research team. We have added a recommendation at the end of our discussion to suggest additional strategies that could further reduce malaria-related morbidity

# Peer review: Reviewer response for version 2

**DOI:** 10.21956/wellcomeopenres.20402.r52580

**Published:** 2022-10-11

**Authors:** Drissa Konaté

**Affiliations:** 1 Malaria Research and Training Center, Bamako, Mali

I approve this version with one comment:

1. Specify SMC campaign rather than mass. 

Is the work clearly and accurately presented and does it cite the current literature?

Yes

If applicable, is the statistical analysis and its interpretation appropriate?

Partly

Are all the source data underlying the results available to ensure full reproducibility?

Yes

Is the study design appropriate and is the work technically sound?

Yes

Are the conclusions drawn adequately supported by the results?

Yes

Are sufficient details of methods and analysis provided to allow replication by others?

Partly

Reviewer Expertise:

Epidemiology

I confirm that I have read this submission and believe that I have an appropriate level of expertise to confirm that it is of an acceptable scientific standard.

# Peer review: Reviewer response for version 1

**DOI:** 10.21956/wellcomeopenres.20025.r52094

**Published:** 2022-09-15

**Authors:** Drissa Konaté

**Affiliations:** 1 Malaria Research and Training Center, Bamako, Mali

**Introduction (needs more detail):**

- Reference: harmonize the reference citations in the main document.
- The author should give more details on study justification because the effectiveness of SMC has already been proven: more cases of malaria are screened despite the SMC?
- Please specify: SMC campaign or mass campaign? If different to SMC campaign,  what approaches of mass campaign was used to administer antimalarial drug.
- SMC is recommended in children up to 10 in Senegal?

**Methods** **(need to clarify some points):**

***Study Site ***

- The rate of test positivity in the general population was 51%: please specify the test (RDT or blood smear).

***Study type, time-period, and population***

- Controls were recruited at concession level based on an apparent good health and a negative RDT.
- Good health: any malaria symptom or any symptom?

**Data management and analysis:**

- The author just needs to specify which tests used and for what comparison: ANOVA, T-test Chi ^2^ Mann, Kruskall.
- What part of the result section were these test were used?

**Results** **(the interpretation of some sentences needs to be clarified):**

- All cases in our study were recruited on the basis of a positive rapid diagnostic test (RDT) and the controls a negative RDT. Thick blood count was positive in 87.80% (CI 95%=81.8-92.39%) of cases and 2.74% (CI 95%=1.45-5.135%) of controls.
- Specify the test used for malaria diagnosis: There was an association between the result of the thick blood test and whether the child was a case or a control (p=1.10 ^-9^). Make this part clearer

**Malaria prevention:**

***Mosquito net***

- Net ownership was much higher among controls (90,85%; CI 95%=87.24-93.52%) than among cases (85,80%; CI 95%=79.7-90.9%) and this difference was not statistically significant (p=0.053).
- Reword this sentence: Net ownership was much higher among controls because it’s not significant.
- (p=0.0000004): also harmonizes p-values.

**Conclusion** **(without aim)**

- It is not necessary to give against the objective of the study.

Is the work clearly and accurately presented and does it cite the current literature?

Yes

If applicable, is the statistical analysis and its interpretation appropriate?

Partly

Are all the source data underlying the results available to ensure full reproducibility?

Yes

Is the study design appropriate and is the work technically sound?

Yes

Are the conclusions drawn adequately supported by the results?

Yes

Are sufficient details of methods and analysis provided to allow replication by others?

Partly

Reviewer Expertise:

Epidemiology

I confirm that I have read this submission and believe that I have an appropriate level of expertise to confirm that it is of an acceptable scientific standard, however I have significant reservations, as outlined above.

# Response

**Published:** 2022-09-16

**Authors:** Isaac Akhenaton MANGA

**Affiliations:** Faculté de Médecine FMPO/UCAD, Senegal

Introduction

- Reference: We have respect the guidelines given by the journal for this part. However if you can give me an example of that I will take in account to improve the presentation of references.
- Justification of the study: You right when you say that the effectiveness of SMC has already shown, but most often it is in the context of operations research, which we have tried to demonstrate by posing the problem. The justification for this study is that the effectiveness of this strategy in mass campaigns has not been evaluated in Senegal. We think we have done this, but remain open to improving this part.
- You right when you say SMC campaign and I'll delete mass campaign in the new version
- SMC is not recommended for children up to 10 years but for those aged 3 months to 10 years. We have therefore amended the sentence in the new version to make it clearer.

Method 

- Study site: The rate of test positivity is for RDT and blood smear and we add it in the new version of this article.
- Study type, time-period and population: an apparent good health means for us without any clinical symptom. We put it in brackets in the new version for more clarifications.
- Data management and analysis: We take in account your suggestions for the test used and we add in the new version in brackets the test and the p-value for each comparaison.

Result

- For the results of the slide reading we have in the new version removed the first sentence. This sentence can be confusing. 
- We have also added for each comparison the statistical test used.

Conclusion

- We have deleted the objective in this part as you have suggested 
